# Identification of novel subgroup A variants with enhanced receptor binding and replicative capacity in primary isolates of anaemogenic strains of feline leukaemia virus

**DOI:** 10.1186/1742-4690-9-48

**Published:** 2012-05-31

**Authors:** Hazel Stewart, Karen W Adema, Elizabeth L McMonagle, Margaret J Hosie, Brian J Willett

**Affiliations:** 1 Medical Research Council-University of Glasgow Centre for Virus Research, Institute of Infection, Immunity and Inflammation, College of Medical, Veterinary and Life Sciences, University of Glasgow, 464 Bearsden Road, Glasgow, UK

**Keywords:** Feline leukaemia virus, FeLV, Anaemia, Receptor

## Abstract

**Background:**

The development of anaemia in feline leukaemia virus (FeLV)-infected cats is associated with the emergence of a novel viral subgroup, FeLV-C. FeLV-C arises from the subgroup that is transmitted, FeLV-A, through alterations in the amino acid sequence of the receptor binding domain (RBD) of the envelope glycoprotein that result in a shift in the receptor usage and the cell tropism of the virus. The factors that influence the transition from subgroup A to subgroup C remain unclear, one possibility is that a selective pressure in the host drives the acquisition of mutations in the RBD, creating A/C intermediates with enhanced abilities to interact with the FeLV-C receptor, FLVCR. In order to understand further the emergence of FeLV-C in the infected cat, we examined primary isolates of FeLV-C for evidence of FeLV-A variants that bore mutations consistent with a gradual evolution from FeLV-A to FeLV-C.

**Results:**

Within each isolate of FeLV-C, we identified variants that were ostensibly subgroup A by nucleic acid sequence comparisons, but which bore mutations in the RBD. One such mutation, N91D, was present in multiple isolates and when engineered into a molecular clone of the prototypic FeLV-A (Glasgow-1), enhanced replication was noted in feline cells. Expression of the N91D Env on murine leukaemia virus (MLV) pseudotypes enhanced viral entry mediated by the FeLV-A receptor THTR1 while soluble FeLV-A Env bearing the N91D mutation bound more efficiently to mouse or guinea pig cells bearing the FeLV-A and -C receptors. Long-term *in vitro* culture of variants bearing the N91D substitution in the presence of anti-FeLV gp70 antibodies did not result in the emergence of FeLV-C variants, suggesting that additional selective pressures in the infected cat may drive the subsequent evolution from subgroup A to subgroup C.

**Conclusions:**

Our data support a model in which variants of FeLV-A, bearing subtle differences in the RBD of Env, may be predisposed towards enhanced replication in vivo and subsequent conversion to FeLV-C. The selection pressures *in vivo* that drive the emergence of FeLV-C in a proportion of infected cats remain to be established.

## Background

Feline leukaemia virus (FeLV) is an important pathogen of both domestic cats and endangered felid species alike [1,2]. There are three main subgroups of FeLV; FeLV-A, B and C; however, there is evidence that only FeLV-A is transmitted efficiently between hosts [1,3]. Although FeLV-B and C are fully replication competent *in vitro*[4,5], replication *in vivo* is thought to require the presence of FeLV-A; thus the A subgroup is commonly referred to as a “helper” virus, being required for transmission and dissemination of FeLV-B and C within the host.

FeLV-A is often mistakenly termed the “low-pathogenicity” variant [6] as approximately 60% of exposed cats mount a competent immune response and successfully clear infection following a transient viraemia [7,8]. However, the pathogenicity of FeLV-A is well documented and in chronically infected cats a range of clinical signs may develop, including immunosuppression, lymphoma and anaemia [9-11]. The disease association and clinical prognosis are influenced by both the genotype of the FeLV-A isolate and the presence of other subgroups which arise *in vivo*[12]. FeLV-B and -C arise within the infected host through either recombination with endogenous retroviral transcripts [13] or through the gradual acquisition of mutations within the viral genome [14], respectively. These viral subgroups are therefore usually identified alongside a concurrent FeLV-A infection [15-17].

FeLV-C is found in approximately 1-2% of chronically infected cats, and its emergence is associated with the development of pure red cell aplasia (PRCA) [9,16,18]. This non-regenerative anaemia is fatal within approximately 2–3 months of FeLV-C arising in the cat [4,19]. The development of FeLV-C from the initial infection with a FeLV-A isolate is accompanied by an alteration in the receptor usage of the virus, from the thiamine transporter THTR1 utilised by FeLV-A [20] to the haem transporter, FLVCR1 utilised by FeLV-C [21]. The binding of FeLV-C to FLVCR1 impairs the normal cellular function of the protein, preventing haem transport in erythroid cells and resulting in a depletion of erythrocyte precursors [22,23]. Although the widespread cellular distribution of both feTHTR1 and feFLVCR1 in the cat means that FeLV-A and -C are able to infect multiple lineages of haematopoietic cells (lymphoid, erythroid and myeloid), the pathogenic potential of subgroup C viruses appears to be conferred by the ability to interfere with the function of FLVCR1 on erythroid progenitor cells, rather than the widespread infection of diverse cell types [24].

The development of either FeLV-B or -C, and the accompanying diseases associated with these subgroups, is due to alterations within the surface unit (SU, or gp70) domain of the envelope glycoprotein gene (*env*) [13,14]. These mutations affect the amino acid sequence of the receptor-binding domain (RBD) within SU, the region of Env that determines the cognate receptor used for cellular entry. Accordingly, mutations within this region of Env alter the cellular tropism of the virus and both FeLV-B and -C possess expanded *in vitro* host ranges [1,25-27]. Previous studies demonstrated that a 241 amino acid region within the Env of prototype FeLV-C (Sarma) conferred the ability to induce PRCA in experimental infections [28]. Subsequently, the primary determinant of this phenotype was mapped more precisely to a string of 92 amino acids within the RBD of isolates of FeLV-C cloned biologically [14,29]. It was noted that there was limited conservation between the sequence of individual isolates of FeLV-C, supporting the assertion that there is minimal inter-host transmission of FeLV-C and that each isolate arises *de novo* within a unique host. Protein signatures or structures that are conserved between all FeLV-C isolates have yet to be identified, and the critical residues that are essential and sufficient to confer FLVCR binding upon Env have not been elucidated.

It has been assumed that the acquisition of the Env mutations that define the C subgroup would lead to the emergence of two distinct viral populations with non-overlapping receptor tropisms within the host, resulting in the FeLV-A/FeLV-C co-infections observed in clinical cases. However, recent studies have suggested that the evolution of FeLV-C *in vivo* may be a gradual process, with viruses displaying intermediate phentoypes and receptor usages co-existing within the infected host. Indeed, virus isolates have since been identified that utilise both THTR1 and multiple FLVCR1 paralogues [30]. Presumably, these FeLV-A/C dual-tropic viruses would eventually give rise to an isolate of FeLV-C that would utilise FLVCR1 solely.

The initial aims of this study were to investigate how specific mutations within FeLV-A Env influence its ability to evolve into dual-tropic variants and to identify the mutations present in dual-tropic FeLV-A/C viruses that confer an expanded receptor usage. This would allow an insight into the sequence of molecular events leading to the development of a highly pathogenic retroviral variant. We utilised primary field isolates of FeLV which had been confirmed previously by receptor interference studies to contain both subgroups A and C. Comparisons between the *env* genes of these subgroup A strains with those of prototypic strains of FeLV would allow identification of mutations which may contribute to an initial interaction with FLVCR1. Their presence would therefore enhance the possibility of FeLV-C development occurring following genetic drift during subsequent cycles of replication. Here, we examine the subgroup A components of primary anaemogenic strains of FeLV and identify amino acid residues that result in an enhanced receptor usage and replicative capacity.

## Results

### Anaemogenic strains of FeLV are composed of heterogeneous viral populations

**Figure 1:**
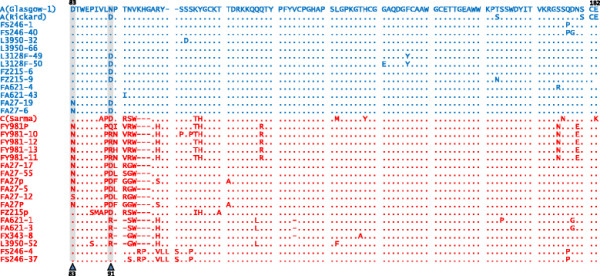
**Sequence alignment of receptor binding domains from the*env*s of anaemogenic strains of FeLV.** FEA cells were infected with primary isolates of FeLV from cats with anaemia. *Env* genes were amplified from total cellular DNA, and the nucleic acid sequences of multiple clones were determined. Sequences in blue were putatively assigned to subgroup A while sequences in red were assigned to subgroup C. Dots indicate conservation with the A (Glasgow-1) sequence. Dashes are included to maintain alignment conservation in the presence of deletion or insertions. Residues 83 and 91 in the N terminal region are highlighted.

The nucleic acid sequences of *env* genes from seven primary isolates of anaemogenic strains of FeLV were determined. Each isolate had been confirmed previously by classical assays for receptor interference as consisting of a mixture of subgroups A and C. Consistent with their designations by interference, multiple unique *env* clones were obtained from each isolate, confirming the heterogeneity of the viral populations within these primary isolates. The majority of variation between Envs was located within the region encoding the RBD (Figure 1). By comparing the amino acid sequences of the novel variants with the prototypic FeLV-A (Glasgow-1) [13] and FeLV-C (Sarma) [4] strains, each Env was tentatively identified as either the subgroup A or subgroup C component of the isolate. Our criteria for classification as a likely subgroup C Env were the presence of substantial amino acid substitutions and/or length polymorphisms in the “Vr1” region [14] of the RBD. Viral variants that appeared genetically to be of the FeLV–B subgroup (present in isolates FY981 and FZ215) due to characteristic recombination events with large stretches of endogenous FeLV sequences were not included in subsequent analyses.

The sequences of the FeLV-A components were highly conserved between isolates, despite the lengthy interim between their isolation. It is widely assumed that all retroviruses acquire significant levels of variation over time due to the error prone enzymatic activity of reverse transcriptase, the actions of cellular antiviral factors such as APOBECs, and the selective pressure from the host immune response. However, while this is indeed the case for the human immunodeficiency virus (HIV), and to a lesser extent the feline immunodeficiency virus (FIV), most isolates of FeLV-A are remarkably similar [4]. In comparison, FeLV-C is thought to be poorly transmissible, and each isolate arises independently within the infected cat. This is reflected by the high degree of divergence we observed in the RBD region of Env between isolates. Despite this heterogeneity, no mutations were identified that were conserved across all the FeLV-C *env* sequences and an essential Env determinant of the FeLV-C phenotype remains to be identified.

Within the A/C mixtures, we observed Envs that were ostensibly subgroup A by sequence alignment and yet bore point mutations from the reference strain (FeLV-A(Glasgow-1)). It can be assumed that the FeLV-A Envs we identified were the parental viruses of the FeLV-C isolates within their respective hosts, therefore we postulated that these mutations may have affected the receptor utilisation of the FeLV-A Env, increasing the likelihood of FeLV-C developing. Within the FA27 isolate, variants were identified with an aspartate to asparagine substitution D83N, a residue that varied in two of the novel FeLV-Cs. This non-conservative mutation (D83N) was documented previously in the FY981 virus [30], a variant that is able to utilise FLVCR1, FLVCR2 and THTR1 [30]. Similarly, an asparagine to aspartate substitution (N91D) was present in three of the novel FeLV-As (L3128F, FZ215 and FA27), ablating a potential site for N-linked glycosylation and aligning with a region of FeLV-C Envs that is critical to the determination of the subgroup C phenotype [4,14]). Asparagine-91 of FeLV-A (Glasgow-1) is replaced with a Serine (S91) in both FeLV-A (3281) and FeLV-A (61E) [6]. In contrast, FeLV-A (Rickard), which has been shown to give rise to FeLV-C *in vivo*[31,32], contains an aspartate residue (D91). D91 is also found in FeLV-C(Sarma) [4] and FeLV-A(945) [33]. As the D83N and N91D substitutions were localised to the primary determinant of FeLV-C receptor usage, we investigated a possible role for these residues in the evolution of FeLV-C from FeLV-A.

### D83N and N91D bearing mutants of FeLV-A (Glasgow-1) are replication-competent

**Figure 2:**
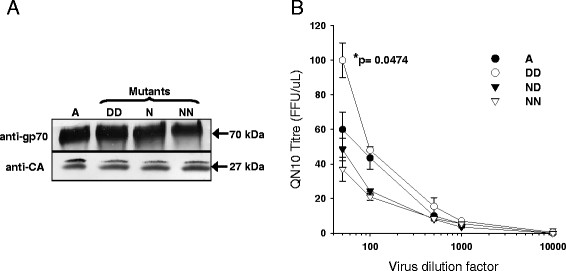
**The DD mutant of FeLV-A (Glasgow-1) displays a higher infectious titre.** (**A**) Three mutants of the FeLV-A (Glasgow-1) molecular clone, possessing mutations at amino acids 83 and(or) 91, were transfected into HEK293T cells. While FeLV-A (Glasgow-1) (A) has the phenotype D83:N91, the mutants DD, ND and NN were D83:D91, N83:D91 and N83:N91 respectively. Viral supernatant was harvested 72 hours post-transfection, filtered and ultracentrifuged, separated by SDS-PAGE, immunoblotted and probed with either anti-gp70 (SU) or anti-p27 (CA). (**B**) FeLV-A (Glasgow-1) (A) or the DD, ND and NN mutants were transfected into 293 T cells and the supernatant was recovered. Serial dilutions of each supernatant were prepared and plated onto QN10 S + L- cells. 72 hours post-infection, foci were scored manually, values represent the mean +/− SEM of two independent experiments.

The wildtype genotype of FeLV-A (Glasgow-1) Env is D83:N91 (DN). Using site-directed mutagenesis, we developed three mutants of the FeLV-A(Glasgow-1) molecular clone, designated FeLV-A (Glasgow-1) D83:D91 (DD), N83:D91 (ND) and N83:N91 (NN). HEK293T cells were then transfected with the functional molecular clones and a chronic FeLV infection of each mutant virus was established. Active infection was confirmed after two weeks in culture by immunoblotting for both viral capsid protein p27 and Env protein gp70 (Figure 2A). During the preparation of virus stocks of the four variants, it was noted that the DD mutant appeared to grow more efficiently in HEK293T cells. In order to measure the approximate titre of the four virus preparations, serial dilutions of the HEK293T-derived viruses were prepared, plated onto QN10 (S + L-) cells, and the number of foci per μl was quantified. This S + L- assay is used widely to confirm the presence of an infectious feline gammaretrovirus [34] and indicated that despite transfection of matched inputs of the four molecular clones into HEK293T cells, the four viruses displayed differences in infectious titre; DD achieved a significantly higher titre than the reference A (Glasgow-1) while ND and NN achieved lower titres (Figure 2B). These observations suggested that the combination of residues D83 and D91 conferred an enhanced replicative capacity upon FeLV-A.

### D83:D91 in Env enhances viral entry mediated by THTR1 and its homologues

**Figure 3:**
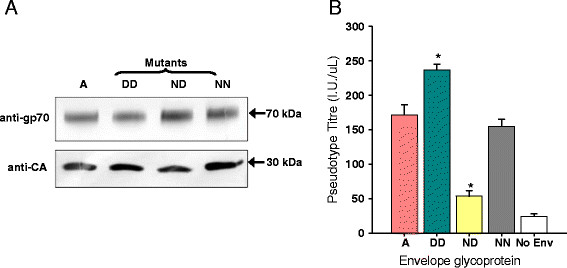
**The DD Env supports more efficient infection of HEK293T cells.** (**A**) MLV(FeLV) *lacZ* pseudotypes were prepared from HEK293T cells. Samples of each pseudotype preparation were pelleted, separated by SDS-PAGE, immunoblotted and probed with either anti-FeLV gp70 or anti-SFFV Gag. (**B**) Matched inputs (RT activity) of MLV(FeLV) *lacZ* pseudotypes bearing the Glasgow-1 (A), DD, ND or NN Envs, were plated onto HEK293T cells. 72 hours post-infection, the cells were stained for expression of lacZ and counted manually. Values are presented as infectious units per μl (I.U./ μl ), mean +/− SE of three independent experiments. The increase in titre noted for the DD mutant and the decrease in titre of the ND mutant (asterisks) are statistically significant in comparison with A (Glasgow-1) (unpaired T test, p < 0.05).

As QN10 assays were designed to detect infectious virus rather than to quantify viral entry, the Glasgow-1 (A), DD, ND and NN *envs* were sub-cloned into a mammalian cell expression vector in order to produce MLV(FeLV) *lacZ* pseudotypes (murine leukaemia virus virions bearing the FeLV Envs and carrying a *lacZ* marker gene), thus facilitating quantification of viral titre based solely on viral entry. Following infection of HEK293T cells with matched inputs of the MLV(FeLV) *lacZ* pseudotypes (Figure 3A), DD Env-bearing pseudotypes yielded a higher titre than those bearing the parent Glasgow-1 Env (Figure 3B). In contrast, ND Env-bearing pseudotypes achieved a lower titre than Glasgow-1 Env-bearing pseudotypes. As the MLV(FeLV) *lacZ* pseudotypes undergo a single cycle of infection, the data suggest that the DD mutation enhanced replication at the stage of viral entry.

**Figure 4:**
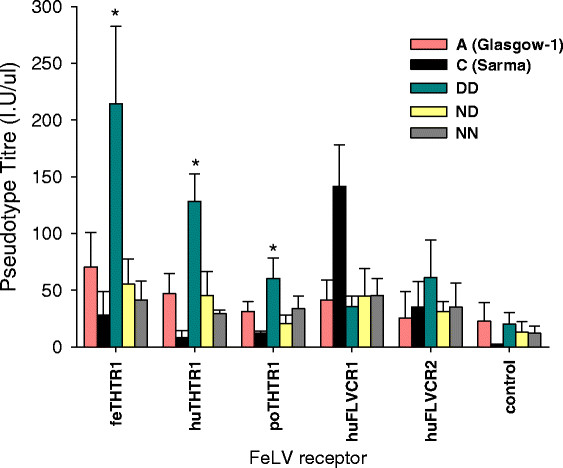
**The DD Env confers enhanced utilisation of THTR1 homologues.** MLV (FeLV) *lacZ* pseudotypes bearing the A (Glasgow-1), C (Sarma), DD, ND or NN Envs were plated onto MDTF cells expressing the feline (fe), human (hu) or porcine (po) THTR1 homologues, human FLVCR1 or human FLVCR2. 72 hours post-infection, cells were stained for expression of lacZ and counted manually. Values represent the mean +/− SEM of three independent experiments. The increase in titre associated with the DD mutation upon feline, human or porcine THTR1 is statistically significant in comparison with A (Glasgow-1) (unpaired T test, p < 0.05).

HEK293T cells are susceptible to all subgroups of FeLV and presumably express all the cognate receptors, although this has never been confirmed directly. Hence, although the DD Env appeared to confer enhanced viral entry, this assay did not allow identification of the receptor(s) being utilised by the virus. Therefore, we repeated the lacZ pseudotype assay using *Mus dunni* tail fibroblast (MDTF) cells engineered to express stably a range of FeLV receptors. Cells were infected with matched inputs (RT activity) of MLV(FeLV) *lacZ* pseudotypes, and the efficiency of infection was quantified. The results indicated that pseudotypes bearing the DD Env displayed a significant increase in the usage of all three THTR1 orthologues in comparison with the wild type FeLV-A (Glasgow-1) Env (Figure 4). In contrast, C (Sarma) Env-bearing pseudotypes displayed a marked preference for hFLVCR1-expressing MDTF cells.

### Enhanced binding of FeLV D83:D91 SU viral receptors

**Figure 5:**
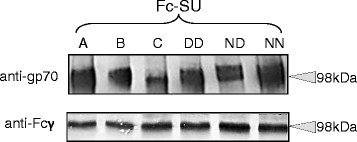
**Expression of soluble Fc-tagged FeLV-SUs.** HEK293T cells were transfected with the pTORSTEN vector into which the SU domains of the Glasgow-1 (A), Gardner-Arnstein (B) and Sarma (C), or the Glasgow-1 mutants DD, ND and NN had been cloned. Matched volumes of supernatant were separated by SDS-PAGE, transferred to nitrocellulose and immunoblotted for both gp70 (4-15% gradient gel, upper) and the human IgG Fc tag (8% linear gel, lower).

**Figure 6:**
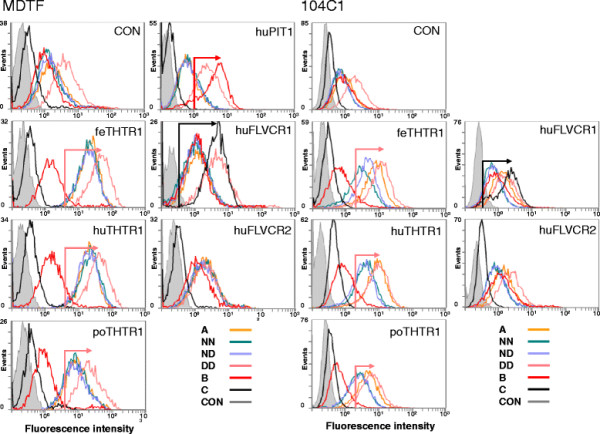
**The DD mutation confers enhanced binding of Fc-SU to multiple receptors.** Matched volumes of supernatant containing the Fc-SU fusion proteins from Glasgow-1 (A), Gardner-Arnstein (B), Sarma (C), or the mutants DD, ND and NN, were added to either MDTF or 104 C1 cells expressing feline, human or porcine THTR1, human FLVCR1 & 2, human Pit1 (MDTF only), or vector only (CON). Fc-SU binding was detected by flow cytometry with PE-conjugated anti-human IgG-Fc. Each histogram represents 10,000 events collected in LIST mode and are representative of two independent analyses. Ordinate displays number of events while abscissa displays fluorescence intensity.

*In vitro* studies with MLV(FeLV) pseudotypes had suggested that the combination of D83 and D91 in Env conferred enhanced viral entry. We asked whether this effect was mediated by increased binding to the viral receptor(s), THTR1 (FeLV-A receptor), FLVCR1 (FeLV-C receptor) and Pit-1 (FeLV-B receptor). Recombinant SU proteins were expressed transiently in HEK293T cells as C-terminal fusions with human IgG-Fc. Immunoblot analysis with anti-gp70 MAb and anti-human IgG Fc confirmed the antigenicity and yield of the SU-Fc proteins respectively, although we were not able to ascertain whether N91 constituted a potential site for N-linked glycosylation (Figure 5). SU-Fc binding to receptor-expressing MDTF (murine) or 104 C1 (guinea pig) cells was then assessed by flow cytometry using matched inputs of SU-Fc protein (informed by immunoblotting of the recombinant SU-Fc proteins with anti-IgG-Fc, see Figure 5) from FeLV-A Glasgow-1 (A), FeLV-B Gardner-Arnstein (B) and FeLV-C Sarma (C) or the FeLV-A Glasgow-1 mutants D83:D91 (DD), N83:D91 (ND) and N83:N91 (NN). The subgroup A-derived A, DD, ND and NN SU-Fc proteins bound to MDTF or 104 C1-expressed THTR1s, B SU-Fc bound to huPit1 expressing MDTF cells (we were unable to derived huPIT1-expressing 104 C1 cells) while C SU-Fc bound most efficiently to human FLVCR1-expressing MDTF or 104 C1 cells (Figure 6), confirming the specificity of the interactions. Each of the Fc-SU fusion proteins displayed a degree of background binding to the control MDTF and 104 C1 cells. Given the widespread expression of the major facilitator superfamily of cell surface molecules, a degree of background binding should be expected. Indeed MDTF cells are known to express both a murine THTR1 homologue and an unidentified accessory molecule that is required for FeLV infection [35]. It was notable, however, that in spite of ensuring that equivalent amounts of each of the Fc-SUs were used in the binding studies, the DD mutant bound more efficiently to both control and THTR1-expressing cells. Each of the subgroup A-derived Fc-SUs bound most efficiently to feline, less so to human THTR1 and weaker still to porcine THTR1, consistent with the cat being the natural host species for FeLV. Of the four FeLV-A SU-Fcs, the DD mutant appeared to display higher binding than the A, NN and ND SU-Fcs to each of the THTRs, suggesting that the DD combination may enhance the Env-receptor interaction, implicating a molecular basis for the enhanced replication (Figure 2) and viral entry (Figures 3 and 4). These findings were mirrored using independently generated cell lines derived from 104 C1 cells. The binding studies with the 104 C1 cells suggested that the NN and ND SU-Fc proteins bound less efficiently to 104 C1-expressed THTR1s than the parental A SU-Fc, suggesting that while mutations such as DD may enhance SU-Fc binding, the converse may be true of NN and ND mutations. As binding of the NN and ND Fc-SUs to THTR1s expressed on MDTF cells was indistinguishable to that of the control A Fc-SU, the data suggest that the cellular milieu in which the receptors are expressed may influence the functionality of the molecule as a viral receptor. Significant weak binding of all SU-Fcs was noted to control MDTF and 104 C1 cells although it was notable that the A and B SU-Fcs had higher background binding than the C SU-Fc protein. It was notable that while the DD SU-Fc bound with a higher efficiency than the other A SU-Fcs to the control MDTF cells, it bound with a similar efficiency to huFLVCR2-expressing cells. As the A, NN, ND and DD SU-Fcs bound to huFLVCR2-expressing cells with similar efficiencies, these data strongly suggest that the enhanced binding of the DD SU-Fc to diverse receptors is a specific property of the DD SU receptor binding domain and not a reflection of variations in the amount of viable SU-Fc in the preparation. Given that the development of FeLV-associated erythroid hypoplasia is marked by a shift in receptor usage from THTR1 to FLVCR, the enhanced binding afforded by the DD mutation may be highly significant in the spread of such variants into compartments expressing the FLVCR receptor; this likely represents the first step towards the biological selection of subgroup C viruses.

### Selective pressures driving the emergence of FeLV-C

**Figure 7:**
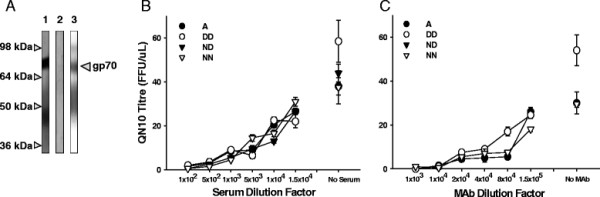
**Neutralisation of FeV by either pooled serum from FeLV-recovered cats or anti-gp70 monoclonal antibody.** (**A**) Purified FeLV-A (Glasgow-1) was separated by SDS-PAGE and transferred to nitrocellulose membrane. Strips of membrane were probed with pooled FeLV-positive sera (lane 1), pooled FeLV-negative sera (lane 2) or murine anti-gp70 MAb (lane 3). 100 I.U. of FeLV-A (Glasgow-1) (A) or the DD, ND and NN mutants were incubated for two hours with serially-diluted pooled (**B**) FeLV-positive serum or (**C**) anti-gp70 MAb, before being titrated on QN10 cells. 72 hours post-infection, foci were enumerated manually. Values represent the mean +/− SEM of two independent experiments.

If, as we predict, the acquisition of substitutions such as N83D and N91D renders the virus more likely to evolve from subgroup A to subgroup C, we may be able to observe this process of evolution *in vitro* by attempting to recreate a similar milieu to that observed *in vivo*. Unfortunately, the site of viral replication and evolution *in vivo* remains unclear, and therefore we can only surmise that it is a site in which both the A and C receptors are expressed. Moreover, additional selective pressures may be placed upon the evolution of the viral Env protein *in vivo* from the adaptive (acquired) immune response of the host. In order to investigate whether the humoral immune response played a role in the evolution of FeLV-C, sera from FeLV-infected cats were pooled and screened for reactivity with gp70. During recovery from infection, cats mount a neutralising response that targets gp70. Accordingly, by pooling sera from recovered cats, we generated a cat polyclonal serum that reacted with gp70 on immunoblot (Figure 7A) and which neutralised infection with FeLV (Figure 7B). When the relative sensitivities of FeLV-A Glasgow-1 and the DD, ND or NN mutants to neutralisation by either the pooled cat serum or a monoclonal antibody targeting gp70 (Figure 7C) were compared, no significant differences were detected, suggesting that the substitutions in amino acids 83 and 91 did not confer resistance to either of these neutralising antibodies. As it is likely that, in individual cats, the response to gp70 may be epitope-specific and that the specificity of the response will vary between cats, we cannot discount the possibility that N83D or N91D containing variants may have either a heightened or reduced sensitivity to neutralisation *in vivo*.

**Figure 8:**
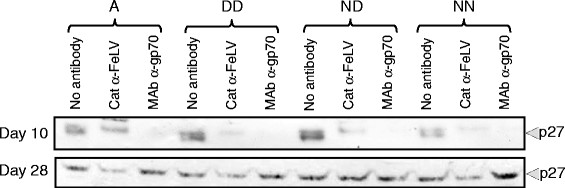
**Replication of FeLV in the presence of sub-neutralising concentrations of anti-FeLV antibodies.** FEA cells were infected with FeLV A(Glasgow-1) (A) or the DD, ND and NN mutants in the presence of sub-optimal neutralising concentrations of either pooled serum from FeLV-positive cats or anti-gp70 MAb. Supernatant was harvested from infected cultures at 10 and 28 days post-infection, concentrated by ultracentrifugation, viral pellets were then separated by SDS-PAGE, transferred to nitrocellulose and stained for capsid protein (p27).

Next, we asked whether culturing each of the viruses in the presence of sub-optimal neutralising concentrations of antibody would promote the emergence of variants with distinct receptor usages. FEA cells were infected with either A (Glasgow-1) or the DD, ND and NN mutants (MOI = 0.01), and a productive infection established. Anti-gp70 MAb or pooled FeLV-positive cat serum was added to the culture medium after the first subculture (72 hours post-infection). Ten days post-infection, the culture supernatants were harvested, concentrated 100-fold by ultracentrifugation, and screened by immunoblot for the presence of viral p27 CA protein. While p27 was detected in the concentrated culture supernatant from cells infected with virus in the absence of antibody at both 10 and 28 days post-infection, both the polyclonal cat serum and, more markedly, the mouse MAb, suppressed virus production at 10 days post infection (Figure 8). As p27 production was detected in all cultures by 28 days post-infection, the data confirmed that we were using a sub-optimal neutralising antibody concentration and that the culture system was biased towards the emergence of neutralisation escape mutants. The cultures were maintained for fifty days in the presence of antibody with routine sub-culture, after which the culture supernatants were harvested from all cultures, concentrated by ultracentrifugation and viral RNA was isolated. *Env* genes were amplified, cloned and their nucleic acid sequences determined. A total of 62 *env* sequences were determined from the 12 cultures (~5 sequences per culture) and were compared with the inocula used for infection.

**Figure 9:**
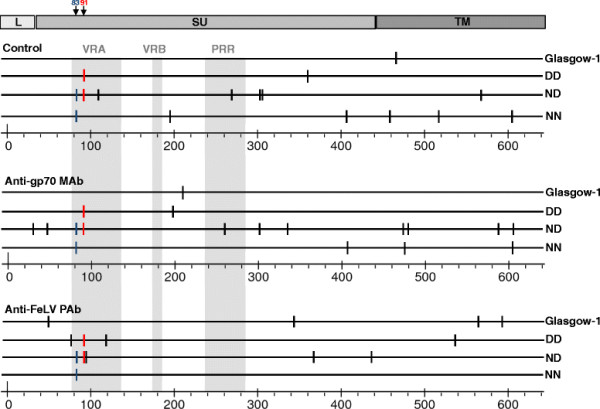
**Acquisition of non-synonymous mutations in the*envs*of FeLV-A mutants following long-term culture.** FEA cultures were infected with FeLV-A (Glasgow-1), or the DD, ND and NN mutants in the presence of pooled feline sera from FeLV-positive cats, anti-gp70 MAbs, or with no antibody (control). 50 days post-infection, *env* sequences were amplified from purified viral RNA, and their nucleic acid sequence were determined.

A total of 59 mutations, 42 of which were non-synonymous,were identified (Figure 9). With rare exceptions, each mutation was identified in a single clone. When comparing the mutations which arose to those described previously or those observed in our original primary isolates (Figure 1), we observed no evidence for the selective expansion of variants with FeLV-C-like sequences. The level of variation varied widely between viruses and while nine non-synonymous substitutions were detected in variants amplified from the culture infected with the ND mutant in the presence of anti-gp70 monoclonal antibody (D30G, T49A, R263K, D305G, T336A, L476P, R482G, M591T, L608P), no substitutions were detected in the culture infected with the NN mutant in the presence of anti-FeLV polyclonal antibody (Figure 9). Mutations were dispersed across both SU (gp70) and TM (p15E) and were not focussed to variable regions A and B (VRA and VRB) or the proline rich region (PRR). The data indicate that under the culture conditions utilised for this study, the four variants had equal propensities to acquire mutations *in vitro* and that the combinations of DN, DD, ND or NN did not alter significantly the likelihood of the Env acquiring non-synonymous mutations.

## Discussion

The amino acid sequence of the receptor binding domain (RBD) of the A subgroup of FeLV varies little between isolates, constraining the virus to usage of the thiamine transporter THTR1 for infection. The switch in subgroup from A to C in cats with pure red cell aplasia is marked by amino acid alterations in the RBD that shift receptor usage from THTR1 to the haem transporter FLVCR1. While all anaemogenic strains of FeLV bear such substitutions, little is known about the genesis of the A to C switch. To date, each isolate of FeLV-C studied has displayed a unique RBD sequence, suggesting that recombination with endogenous *env* sequences is an unlikely source of the mutated RBD. In contrast, the emergence of subgroup B viruses is associated with recombination between exogenous and endogenous FeLV *env* sequences [13,36,37]. A more likely mechanism for the derivation of subgroup C viruses would be the acquisition of mutations or deletions *in vivo* in response to a selective pressure from the host, either through pressure to escape the adaptive immune response or through receptor availability in the tissue in which the virus replicates. Such a mechanism predicts the presence of variants with an intermediate tropism; subgroup A viruses with point mutations in the RBD that confer an enhanced or expanded receptor usage. Previous studies identified a subgroup C virus, FY981 that had retained the ability to utilise the subgroup A receptor THTR1 for infection [30], confirming that there are indeed “dual-tropic” or “poly-tropic” viruses amongst primary isolates of anaemogenic strains of virus. FY981 is actually a poly-tropic virus as it is able to utilise a third receptor (FLVCR2) in addition to THTR1 and FLVCR1 [30]. Here, we demonstrated that subtle variations in the RBD of subgroup A viruses may have significant effects on the way the viruses interact with their receptors, potentially predisposing the viruses to *in vivo* mutagenesis. Accordingly, the presence of the combination of D83 and D91 in the background of A (Glasgow-1) was sufficient to enhance receptor binding, viral entry and viral replication. Residue D91 is particularly intriguing as it is present in the well-characterised Rickard strain of FeLV. In two separate studies examining recombination in FeLV infection, it was noted that inoculation of cats with a molecular clone (pFRA) of the Rickard strain of FeLV resulted in 1 of 3 [31] and 1 of 5 [32] cats developing an FeLV-C associated anaemia. As FeLV-C is thought to arise in <1% of infected cats, the high incidence of FeLV-C emergence following inoculation with FRA (33% and 20% respectively) may suggest an enhanced propensity for the development of FeLV-C. Mechanistically, a scenario can be envisaged whereby some subgroup A viruses may be inherently more pathogenic than others due to an enhanced ability to infect and spread in the infected host, a feature determined largely by the affinity of the Env for the viral receptor. Indeed, such a virus (FeLV-945) has been described and shown to have a higher binding affinity for its receptor [38]. Mapping the determinants of the enhanced binding of the FeLV-945 SU to feline cells suggested that the major determinant of the enhanced binding of the 945 SU resided in variable region B (VRB) of gp70. FeLV-945 is a D83:D91 virus, similar to the DD mutant we examined in this study; however, inserting the VRA of 945 in the background of FeLV-61E (an SU that binds with a lower affinity to its receptor) did not confer an enhanced binding upon the 61E SU, suggesting that multiple determinants in gp70 may contribute to the receptor binding affinity. However, it should also be noted that the 61E SU varies from the Glasgow-1 SU at a number of other residues across gp70; the context in which D83:D91 is expressed may be critical to its effect on binding affinity. Moreover, in this study, we expressed SU fusion proteins with a C-terminal IgG Fc-tag (dimeric) and binding was assessed on both mouse and guinea-pig cells expressing individual receptors, whereas the 945-SU proteins [38] were expressed as C-terminal HA tagged proteins (monomers) and binding assessed on the feline lymphosarcoma cell line 3201, a cell line that produces a soluble 35 kDa endogenous FeLV Env protein capable of viral interference [39]. Such experimental differences may modulate both the affinity and the specificity of the Env-receptor interaction in the two systems. For example, it has been shown that the context in which the receptor THTR1 was expressed altered the efficiency of receptor usage by FeLV [30] while soluble endogenous FeLV Env produced from 3201 cells conferred infectivity on the otherwise defective FeLV-T Env [40]. Irrespective of the differences in the experimental systems, the enhanced binding of the Glasgow-1 DD mutant SU-Fc to THTR1 was consistent with the enhanced entry and replication of the virus, while the high affinity binding of the 945-SU was consistent with enhanced binding of intact virus particles from FeLV-945 to the same cells [38].

It is possible that both the affinity of the FeLV SU for its cognate receptor and its ability to induce fusion once bound combine to determine the eventual route of viral entry. Our data may predict that during FeLV-C evolution, additional mutations accumulate during long-term viral replication and that these mutations decrease SU affinity for THTR1 whilst increasing the relative affinity for the FLVCR1 homologues. Such viral evolution would eventually result in an Env capable of mediating fusion and entry via FLVCR1, producing the FeLV-C phenotype and associated PRCA symptoms. This theory is supported by the observation that FeLV-C (Sarma) displayed a severely limited ability to bind to THTR1 despite possessing the D83:D91 motif. However this altered binding affinity may be mediated by a range of mutations across the SU, not comprising a single binding motif, explaining why individual FeLV-C Env proteins are functionally but not genetically conserved. It is possible that the overall final Env conformation, rather than specific individual residues, permits FLVCR1-mediated membrane fusion and cellular entry. The deletions which we observed in multiple FeLV-C *env* clones from our primary isolates may be essential for decreasing the affinity of the THTR1-SU interaction and allowing FLVCR1-mediated entry. This deletion may represent the final mutation in the progression from FeLV-A to -C. The mechanism(s) underlying the deletion of residues within the RBD-encoding region of *env* remain to be ascertained; however, a scenario could be envisaged wherein the RNA structure within some regions may be less stable and inherently more likely to break and reform.

Having established that subtle variations in the VRA of FeLV-A could have a significant impact upon the biological properties of subgroup A FeLV Glasgow-1, we asked whether we could mimic the *in vivo* selective pressure exerted upon FeLV by the humoral immune system. By culturing virus in the presence of sub-optimal concentrations of either monoclonal anti-gp70 antibody or pooled serum from FeLV-infected cats, we demonstrated the acquisition of non-synonymous mutations with time in culture, although we were not able to demonstrate a shift from subgroup A (THTR1-using) to subgroup C (FLVCR1-using). Subgroup C viruses emerge in an estimated 1% of anaemic cats, suggesting that a relatively rare set of circumstances combines to drive their evolution. It is possible that the epitope specificity of the antibody response elicited following infection may prove critical in determining the composition of variants that evolve, and so serum from cats from which FeLV-C had been isolated rather than a diverse pool of FeLV-infected cat sera would be required to mimic this response *in vitro*. While our working hypothesis is that the humoral immune response influences the likelihood of FeLV-C emerging in infected cats, other factors may have a significant impact upon viral evolution; for example VRA may constitute a T cell epitope in some cats and pressure to escape a cellular immune response may play a role in driving variation in VRA. Alternatively, variation amongst cats in both the levels of receptor expression and the cell types upon which the receptors are expressed may determine sites of viral replication. A scenario could be envisaged whereby co-expression of both THTR1 and FLVCR in the same cell may permit the emergence of variants with dual tropisms and ultimately a specific tropism for FLVCR1-expressing cells.

A detailed analysis of the mutations that were identified allowed several inferences to be made. Firstly, the rate of mutation appeared consistent across all cultures, indicating viral genetic drift occurred at similar rates regardless of the presence or absence of VNAs. Additionally, few mutations were identified more than once, indicating a single FeLV genome had not emerged as the dominant viral species in any culture. However, the limited capacity of our *env* selection method (cloning as opposed to deep-sequencing) must be taken into account as it is possible that the amplification of more *env* sequences, or the use of alternative techniques, may have produced different results. Amplification and cloning of *env* genes may not provide a sufficiently broad picture of the genomes present in the culture. It remains possible that dual-tropic virus strains, or those containing mutations indicative of a FeLV-C phenotype, were present in some cultures, but were not detected during our analysis. The results presented herein therefore represent a “snapshot” of the viral genomes present at 50 days post-infection. We were unable to determine whether specific viruses formed prominent subpopulations during the course of infection, indicative of a species with a replicative advantage expanding.

Apart from viral genetic drift, there are numerous alternative mechanisms for the observed mutations; for example, some mutations may have arisen at sites as a result of structural biochemistry. There is evidence that adenine-thymine tracts (consisting of four consecutive A or T nucleotides) are associated with “bends” in the nascent DNA strand, and are more likely to be mutated through misincorporation [41]. As 13 of the 59 mutations (~22%) observed in the long-term replication study occurred in AT-rich tracts (nucleotide data not shown), it is possible these are due to this phenomenon, rather than either neutralisation escape or receptor tropism expansion as originally predicted. In addition, some mutations may have arisen as the result of apolipoprotein B mRNA-editing enzyme catalytic polypeptide (APOBEC) activity. APOBECs mutate retroviral genomes by deaminating the cytosines within DNA, eventually causing an accumulation of G to A mutations in the RNA genome. However, there was minimal evidence of APOBEC activity in the *env* sequences analysed in this study; of the 59 mutations identified only 7 were G-to-A transitions. Additionally, only 3 of these 7 were found within likely targets for feline APOBECs (AGG or GGG motifs) [42]. This is in accordance with other reports that APOBECs exhibit weak restriction of FeLV in natural infections [42,43].

The inclusion of VNAs in our long-term replication study was based upon the assumption that FeLV-C evolution may be a result of the replicating virus escaping antibody-mediated neutralisation. This hypothesis is not without precedent as there are numerous instances of retroviral antigenic variation and receptor usage alterations being driven by pressure from the host immune response. VNA play a role in the selection and expansion of viral variants in both simple and complex retroviruses, and this has been mimicked successfully *in vitro* in numerous cases [44-46]. In the example of equine infectious anaemia virus (EIAV), an *in vitro* model of viral evolution found that 13 viral passages were required to obtain an antibody-escape mutant. This phenotype was conferred by only two altered epitopes in the SU domain [45]. This is a similar timespan to that used in our study, indicating it was sufficient to observe antibody-escape mutants. Despite the lack of FeLV-C associated mutations observed here, development of FeLV-C as a consequence of antibody escape remains a plausible theory. There are numerous variables in this process which could not be replicated in our model, including the broad range of antibodies produced in a competent immune response. Before definitive conclusions can be drawn about the role of VNAs in FeLV-C development, this experiment should be repeated using a more extensive range of both polyclonal and monoclonal antibodies.

## Conclusions

Primary isolates of FeLV-A and C consist of heterogeneous viral populations, and the subgroup A components of these isolates display a range of subtle mutations in Env. In the context of FeLV-A Glasgow-1, the combination of D83 and D91 in gp70 allowed increased binding to THTR1 and enhanced both viral entry and replication. Such properties may predispose viruses to evolution from subgroup A to subgroup C by enhancing spread of the virus into cellular compartments where ability to use FLVCR is selected preferentially. These data provide a first step towards elucidating why FeLV-C emerges infrequently in infected animals and provide further evidence that despite a high degree of genetic homogeneity, not all FeLV-As are equal.

## Methods

### Cell Lines and plasmid constructs

*Mus dunni* tail fibroblast (MDTF) cells (ATCC Catalogue CRL-2017) and guinea pig fibroblast (104 C1) cells were maintained in low-glucose DMEM (Gibco), supplemented with 10% foetal bovine serum, 100U/mL penicillin and 100 μg/mL streptomycin. Human embryo kidney (HEK293T) cells [47], feline embryo A (FEA) cells [26] and QN10 (S + L- feline embryonic fibroblastic AH927) cells were maintained in high-glucose DMEM (Gibco) supplemented with 10% foetal bovine serum, 100U/mL penicillin and 100 μg/mL streptomycin. The pMFG plasmid [48], encoding β-galactosidase (lacZ) with a MLV-packaging signal, was used to detect pseudotype entry and retroviral integration through expression of the lacZ reporter gene. The pCMVi plasmid [49] encodes MLV *gag-pol* and was used to produce MLV-packaged pseudotypes. Retroviral expression vectors based upon the pFB-NEO plasmid (Agilent Technologies) containing the complete cDNA of various retroviral receptors with 5’ haemaglutinnin (HA) tags were kindly provided by Prof C. Tailor, University of Toronto [50,51]. The pVR1012 plasmid is a eukaryotic expression vector modified from the pUC plasmid [52] containing a cytomegalovirus early promoter to drive target gene expression. The pTORSTEN expression vector drives expression of soluble proteins with an incorporated C-terminal human IgG1-Fc tag [53].

### Molecular cloning of proviral *env* genes from primary isolates

Feline embryonic fibroblast (FEA) cells were infected with seven primary FeLV isolates from the United Kingdom, isolated from 1977 to 1994. The FEA cell line is permissive to all three subgroups of FeLV and therefore would not be expected to restrict the growth of any viral variants. The seven isolates had been classified previously by interference assays [17] and found to be FeLV-A/C co-infections. Two isolates also contained FeLV-B (A/B/C mixtures). Successful infection and viral release from the FEA cells was confirmed by a enzyme-linked immunosorbent assay (ELISA) for the viral capsid protein p27 (data not shown). Proviral *env* sequences were amplifed from purified genomic DNA by using the polymerase chain reaction (PCR) using primers based upon published exogenous strains of FeLV. The amplified *envs* were cloned into the pCR2.1 vector (Invitrogen Life Technologies Ltd., Paisley UK) and the nucleic acid sequence determined using the BigDye Terminator v1.1 Kit (Applied Biosystems, Paisley, U.K.) followed by analysis on using an ABI Prism 3130xl Genetic Analyser (Applied Biosystems). Nucleic acid sequences and their predicted amino acid translations were processed using Accelrys Gene 2.5. Nucleic acid alignments were generated using ClustalW and MEGA5.

### Stable transduction of MDTF and 104 C1 cells

VSV-G-enveloped pseudotypes encapsulating transcripts of the pFB-NEO vector, encoding cDNAs of the desired receptor, were produced by transient transfection of HEK293T cells. Cells were transfected with Superfect Reagent (Qiagen, U.K.) according to the manufacturer’s protocol. 72 hours post-transfection, pseudotypes were harvested, passed through a 0.45 μm filter, and used to infect 1x10^5^ adherent cells. 72 hours post-infection the stably-transduced cells were selected by G418 treatment (800 μg/mL). Expression of the retroviral receptor was confirmed using immunofluorescence directed against the C-terminal HA tag.

### Site-directed mutagenesis

The QuickChange Lighting Mutagenesis kit (Agilent Technologies, U.K.) was utilised to mutate the D83 and N91 residues of the FeLV-A(Glasgow-1) molecular clone. The protocol was conducted according to manufacturer’s instructions; however PCR products were precipitated with ethanol/sodium acetate following Dpn1 treatment. They were then resuspended in a small volume of water prior to transformation into competent cells. The sequence of mutagenesis primers is available upon request.

### Neutralising antibody preparations

Neutralising murine monoclonal antibody 46II F2 D10 F4 I3 against the FeLV gp70 was obtained from Prof. H. Lutz. It is not known which epitope within gp70 the MAb targeted. Serum samples from FeLV naturally-infected, recovered cats were kindly donated by the Companion Animal Diagnostic Unit, University of Glasgow. Their assays, being virus isolation and capsid ELISAs, had confirmed the sera were all FeLV virus-negative and antibody positive. Positive sera were pooled and used in virus neutralisation and long-term viral replication assays.

### Immunoblotting

Immunoblots were performed to detect expression of specific proteins in both feline sera and concentrated virus preparations (prepared by centrifugation of cell-free virus supernatant, at 30 000 rpm for one hour in a Beckman Ultracentrifuge with a SW40 rotor). Samples were diluted to the desired volume using bromophenol blue protein loading buffer and heated to 90°C for five minutes prior to electrophoresis. Electrophoresis was conducted in 4-12% gradient precast polyacrylamide gels (Invitrogen) at 90 V for two hours. Proteins were transferred to nitrocellulose membranes using the iBlot transfer system (Invitrogen). Membranes were blocked overnight using 2% skimmed milk powder and 0.1% Tween-20. Primary antibodies against the CA proteins (VPG19.1 anti-FeLV p27 or R187 anti-SFFV p30) were used at a dilution of 1:500, whereas the anti-gp70 MAb was used at 1:10^4^ in blocking buffer. Biotinylated secondary antibodies from various species were utilised (Vector Laboratories, U.K.) at a 1:1000 dilution in blocking buffer. An alkaline phosphatase-labelled avidin complex was then bound to the membrane (ABC-AmP kit, Vector Laboratories), before proteins were visualised using a chromogenic alkaline phosphatase substrate.

### QN10 focus-forming assays

QN10 (S + L-) cells were seeded into 6 well plates at 20% confluency and allowed to adhere overnight prior to infection. Infectious virus was produced by transient transfection of HEK293T cells with molecular clones based upon the FeLV-A (Glasgow-1) construct. Viral supernatant was harvested and used to infect the cells for two hours at 37°C. 72 hours post-infection, the numbers of foci were manually counted and the viral titre (in FFU/μL) was calculated.

### LacZ staining

Pseudotypes containing the Env proteins of interest and encapsulating transcripts of the pMFG plasmid were used to infect both HEK293T and MDTF cells. 72 hours post-infection, lacZ expression was measured by 5-bromo-4-chloro-3-indolyl-β-d-galactoside (X-Gal) staining. Briefly, confluent monolayers of cells were washed in PBS before being fixed with 0.5% glutaraldehyde for 30 minutes. Fixative solution was removed and cells re-washed before the addition of staining solution (PBS containing 0.02% X-Gal, 3 mM ferro-cyanide, 3 mM ferri-cyanide, 1.3 mM MgCl_2_). Plates were stored at 4°C overnight before the lacZ-expressing cells (indicated by a blue appearance) were manually counted.

### Cell-binding assays with soluble SU proteins

The pTORSTEN expression vector was used to express soluble Fc-SU fusion proteins with an incorporated C-terminal human IgG1-Fc tag [53]. The SU domains of all Env proteins were subcloned into pTORSTEN, HEK293T cells were then transiently transfected using polyethylenimine (Polysciences Inc., U.K.) and the supernatant containing the protein of interest was harvested 3 days post-transfection and passed through a 0.45 μm filter. Expression of soluble proteins was confirmed by immunoblotting using both anti-gp70 and anti-Fc. For cell-binding assays, MDTF and 104 C1 cells were dispersed using 1 mM EDTA before 50μL of protein-containing supernatant was incubated with 10^5^ cells for 30 minutes on ice. Cells were washed with PBS containing 1% bovine serum albumin and 0.1% sodium azide (PBS-BSA-Az). 0.5 μg of PE-labelled antibody specific for human IgG (eBiosciences, U.K.) was then added to each reaction and incubated for a further 30 minutes on ice. Cells were washed and resuspended in PBS-BSA-Az before before analysed on a Beckman Coulter Cytomics FC500 flow cytometer.

### *Env* cloning from viral RNA

Viral supernatant from chronically infected FEA cultures was harvested and passed through a 0.45 μm filter, prior to ultracentrifugation at 30 000 rpm for one hour at 4°C. Viral pellets were re-suspended overnight at 4°C in PBS. Viral RNA was extracted using the QIAamp UltraSens Viral kit (Qiagen) according to manufacturer’s instructions. Following elution with water the RNA was DNase-treated to remove potential contaminating cellular gDNA (Amplification-grade DNase, Invitrogen). DNase was inactivated by EDTA-treatment and heating the sample to 65°C for 15 minutes. First-strand cDNA synthesis using oligo-dT primers was conducted with a commercial MLV RT enzyme (Invitrogen) according to manufacturer’s instructions. Ribonuclease inhibitors (RNaseOUT, Invitrogen) were included in all cDNA synthesis preparations. Oligonucleotide primers for amplification of *env* sequences were obtained from MWG Biotech (Ebersberg, Germany); primer sequences are available upon request. All PCRs were conducted using a commercially available mastermix (High Fidelity PCR Master, Roche, U.K.). Amplicons were cloned into the pVR1012 vector and the inserted *env* genes were sequenced using the BigDye Terminator v1.1 Cycle Sequencing kit (Applied Biosystems, U.K.) according to manufacturer’s instructions. Sequencing reaction products were analysed using an ABI Prism 3130xl Genetic Analyser (Applied Biosystems) and data analysed using the DNA Dynamo (Blue Tractor Software Ltd, U.K.) and SeaView software [54].

## Competing interests

The authors declare no competing interests.

## Authors’ contributions

KWA conducted the primary isolate infections, *env* cloning and sequencing analysis. ELM conducted the *lacZ* pseudotype assays upon HEK293T cells. HS conducted all remaining experiments. BJW and MJH conceived the study and designed the experiments. HS and BJW prepared the manuscript while all authors read and approved the final manuscript.
